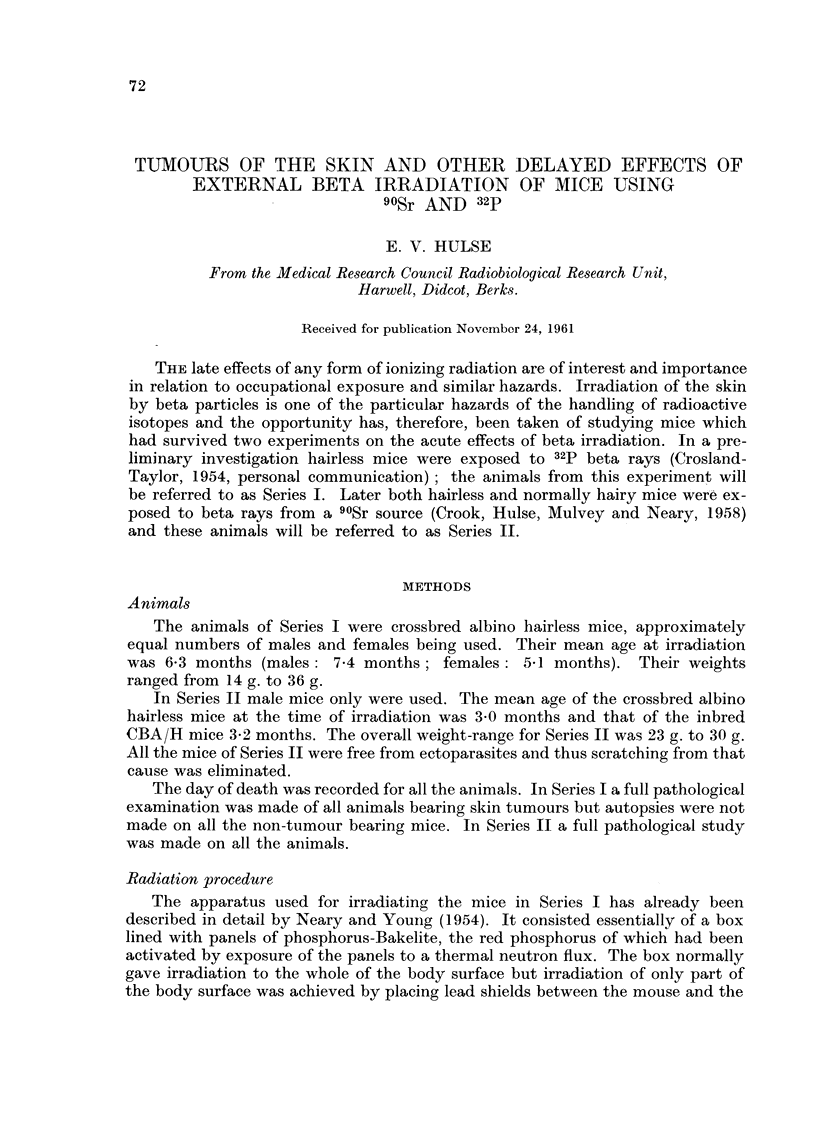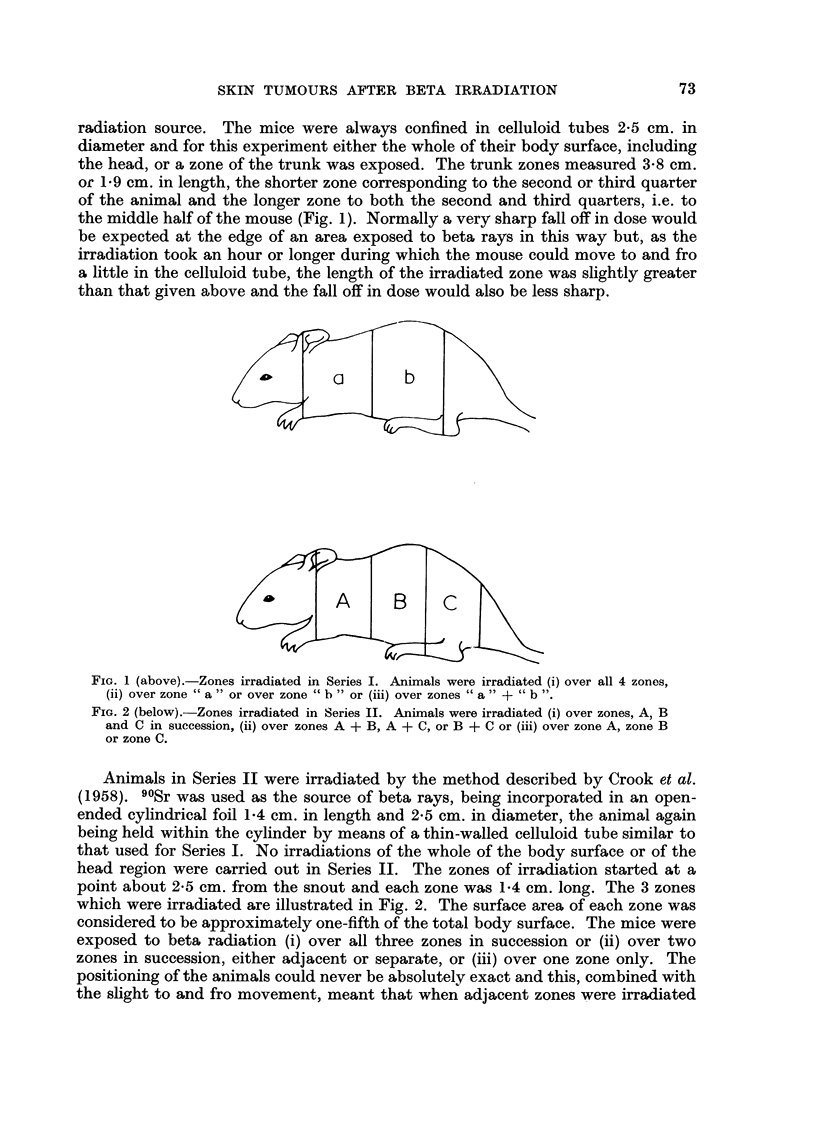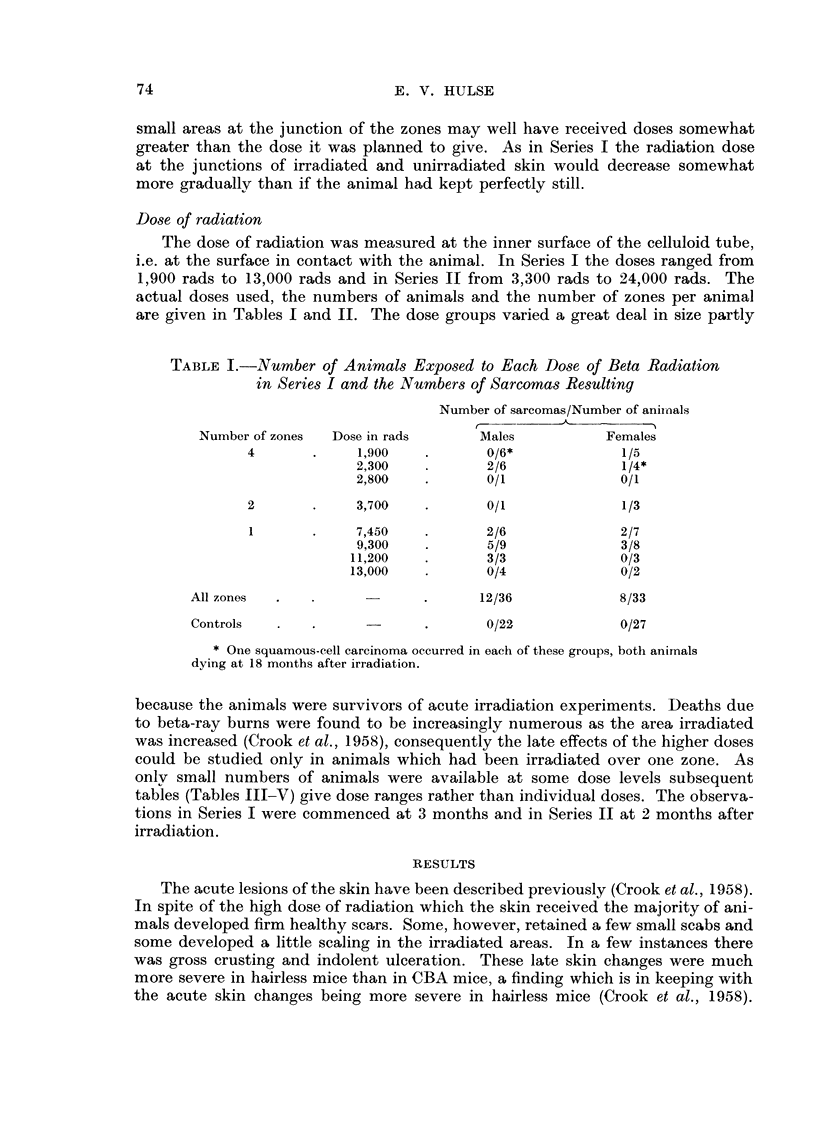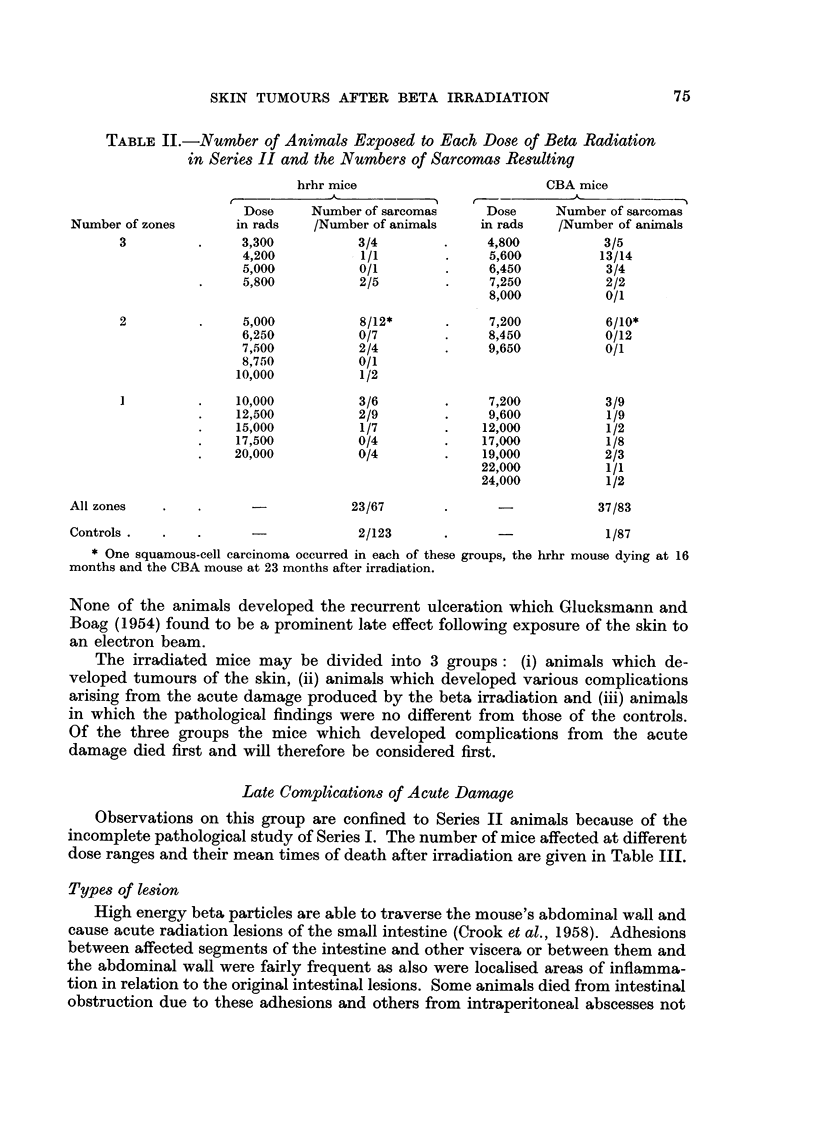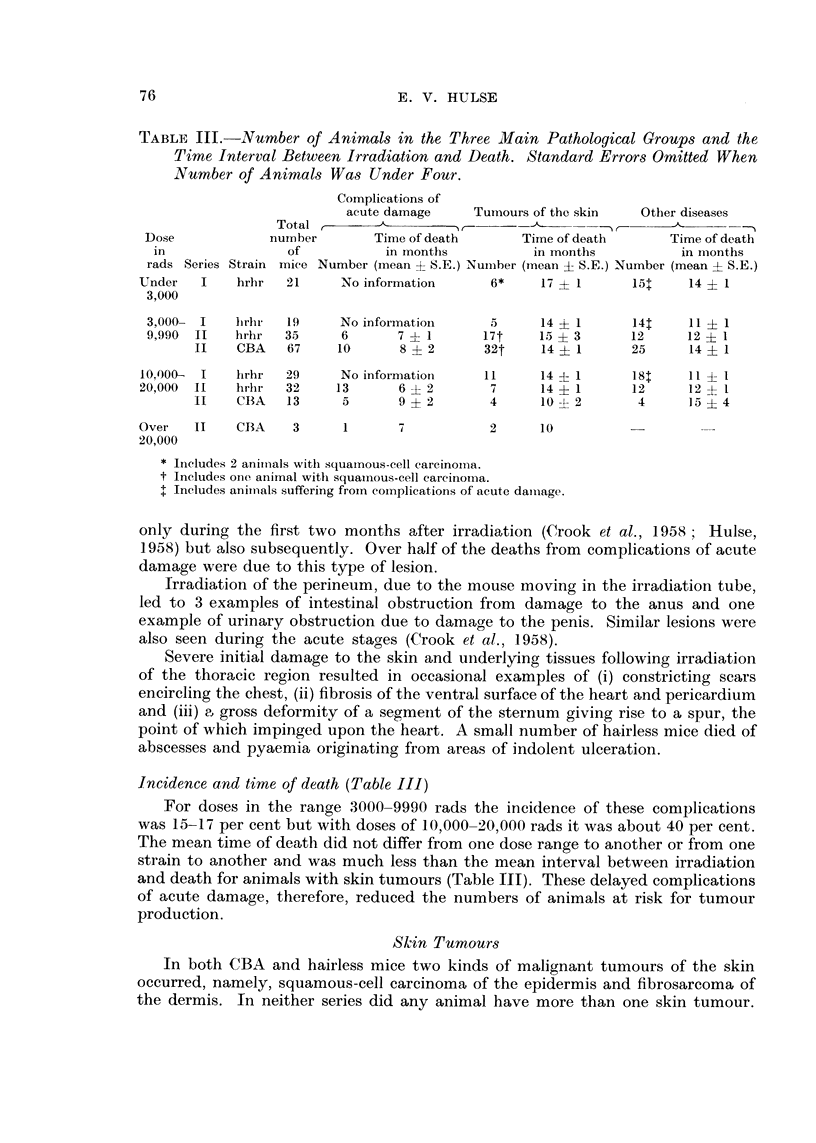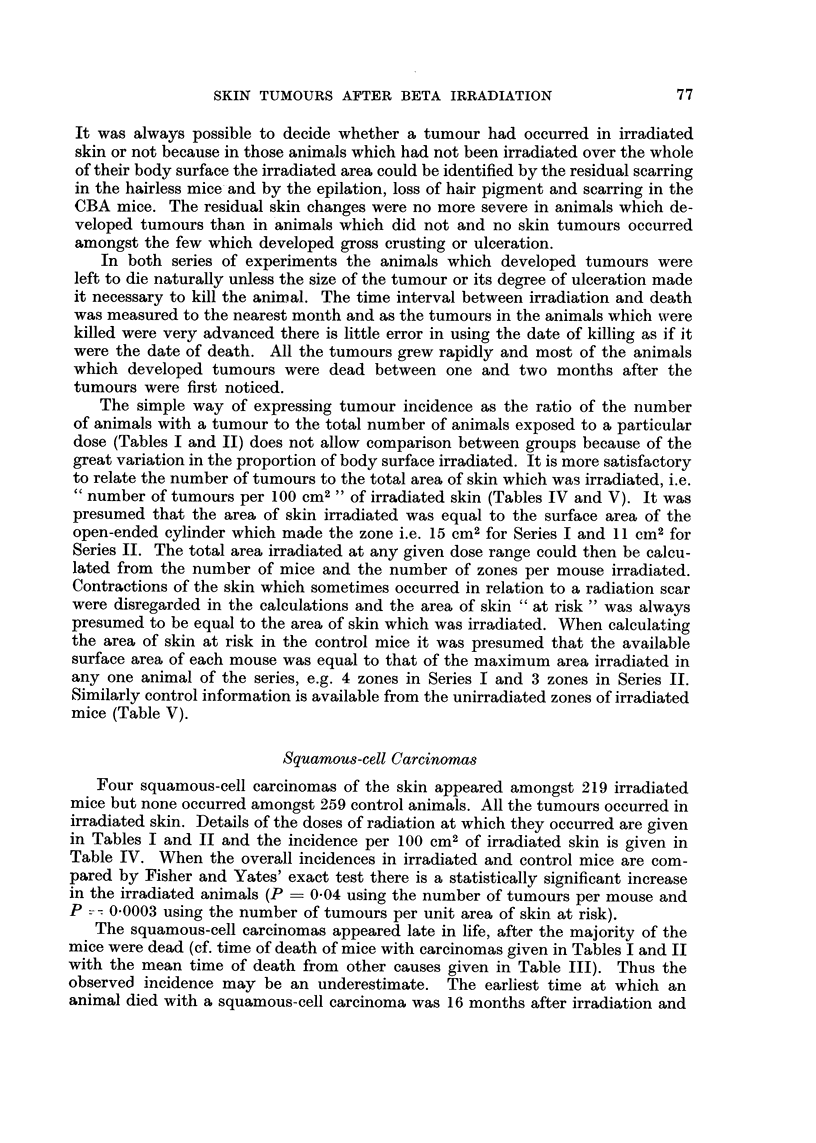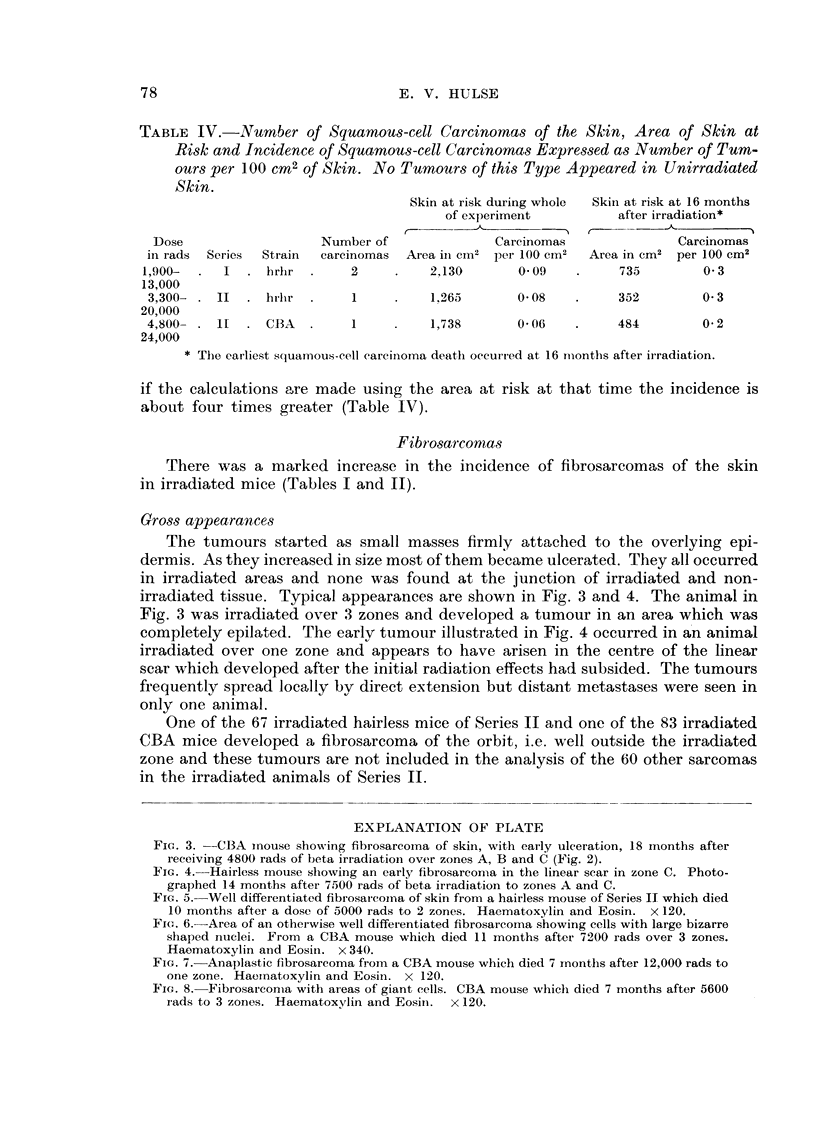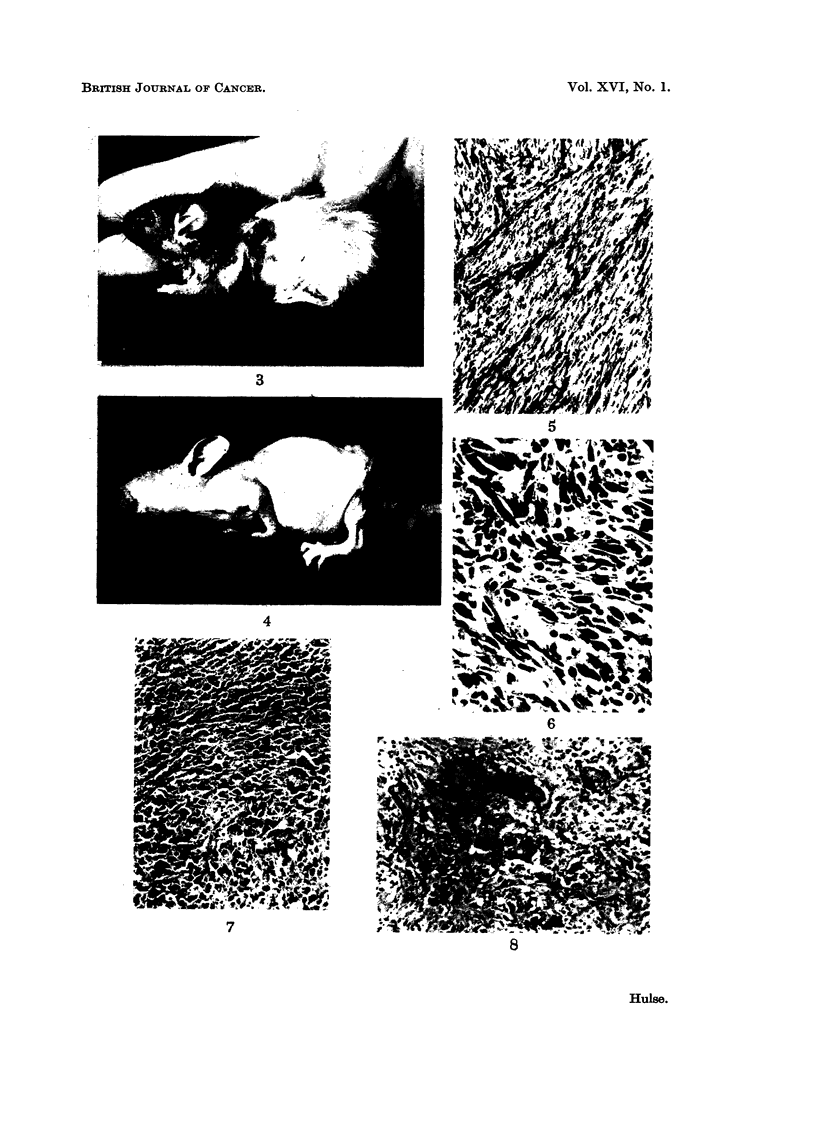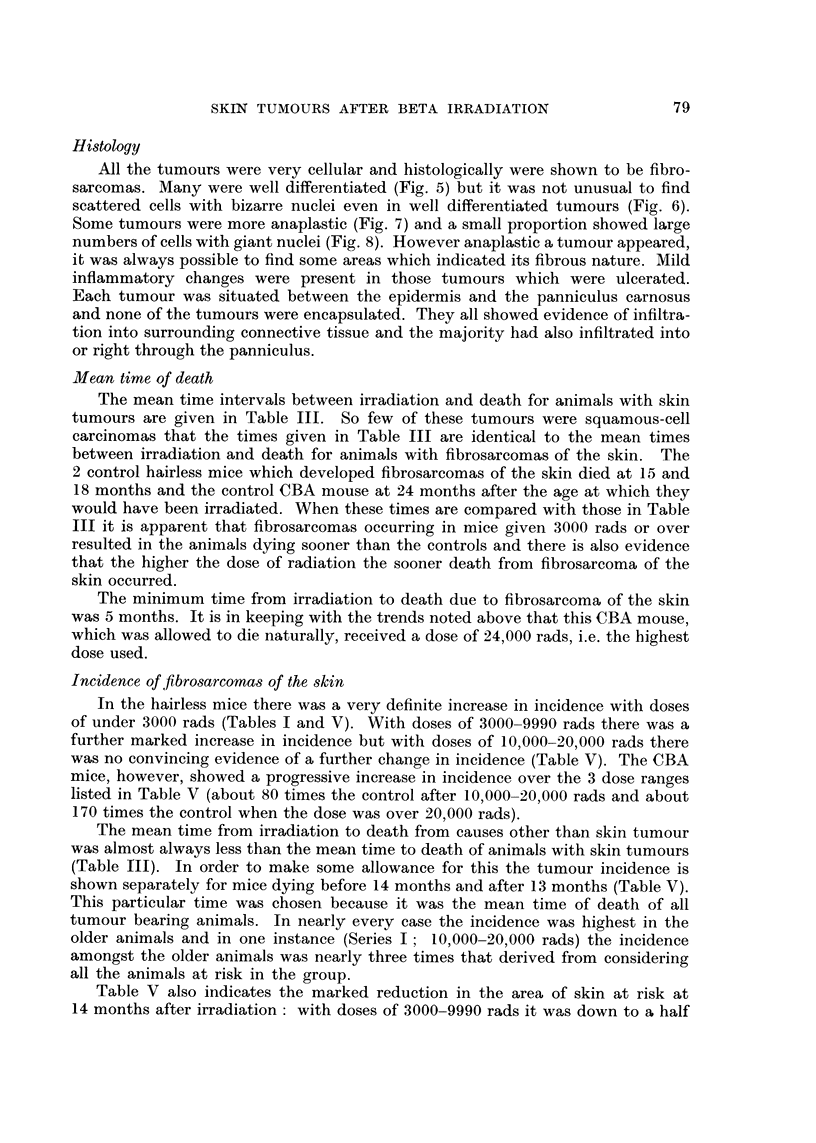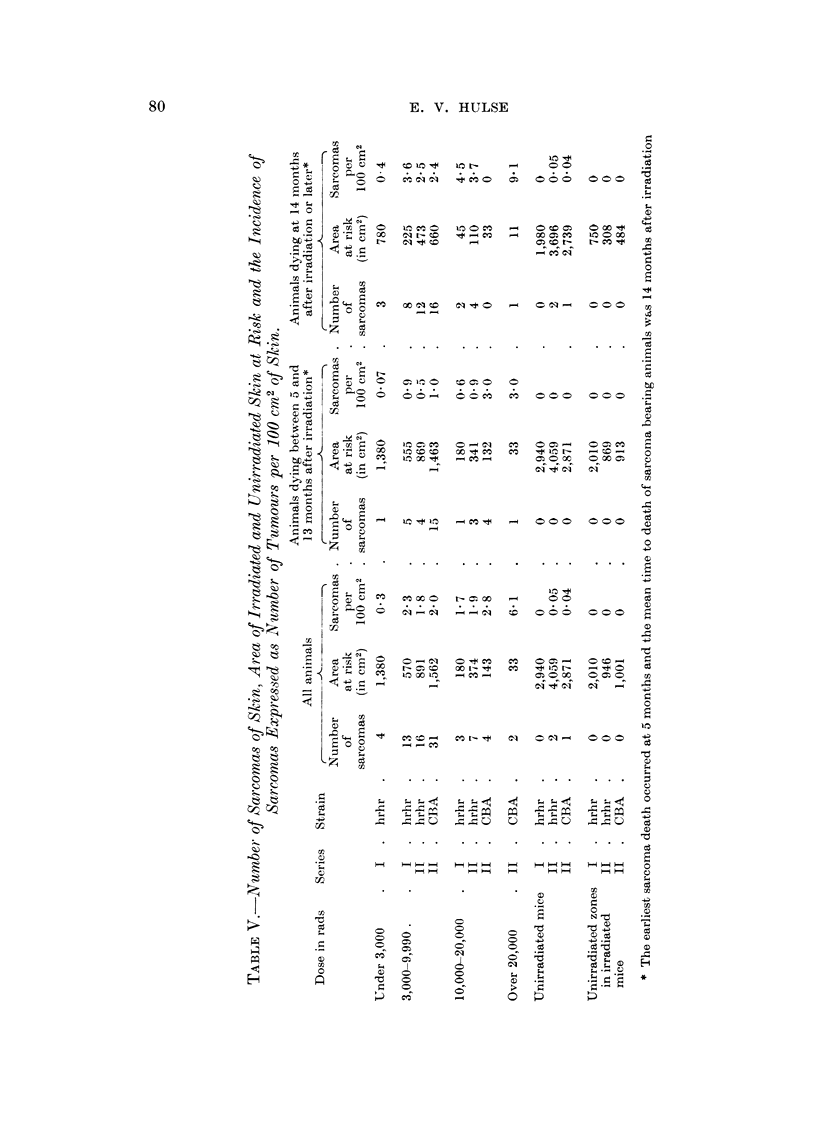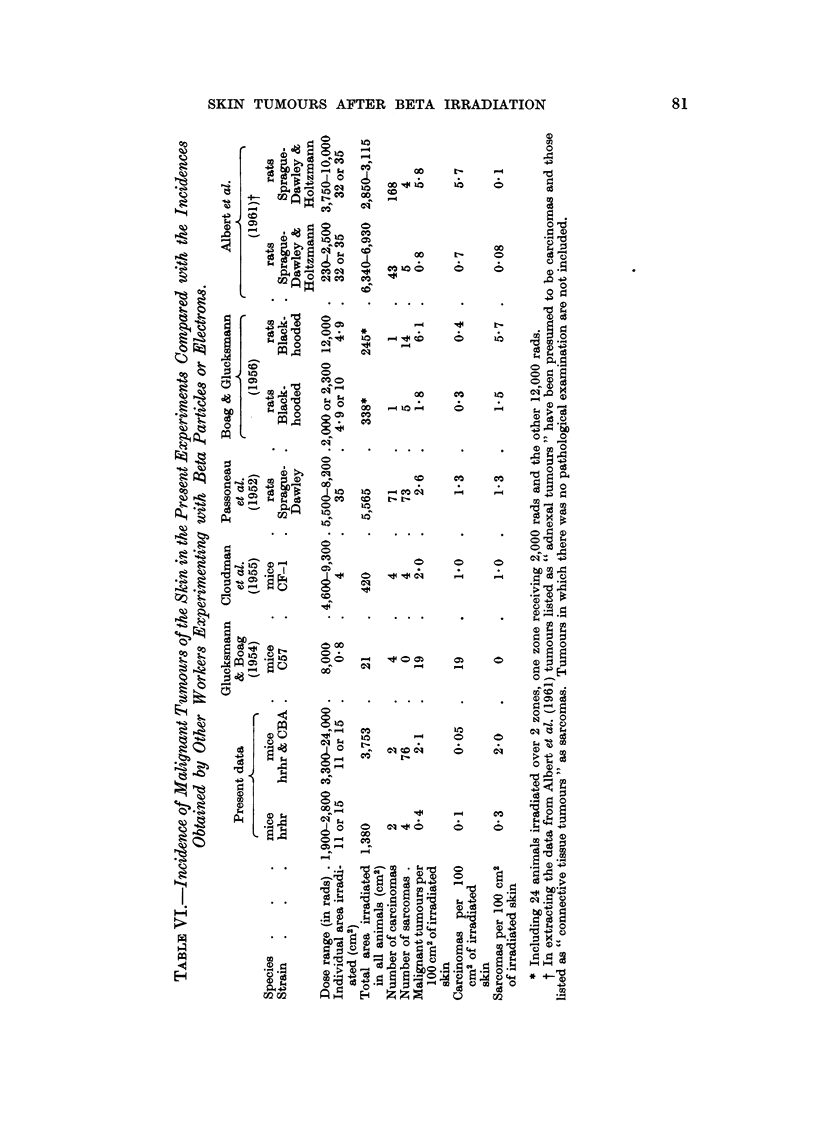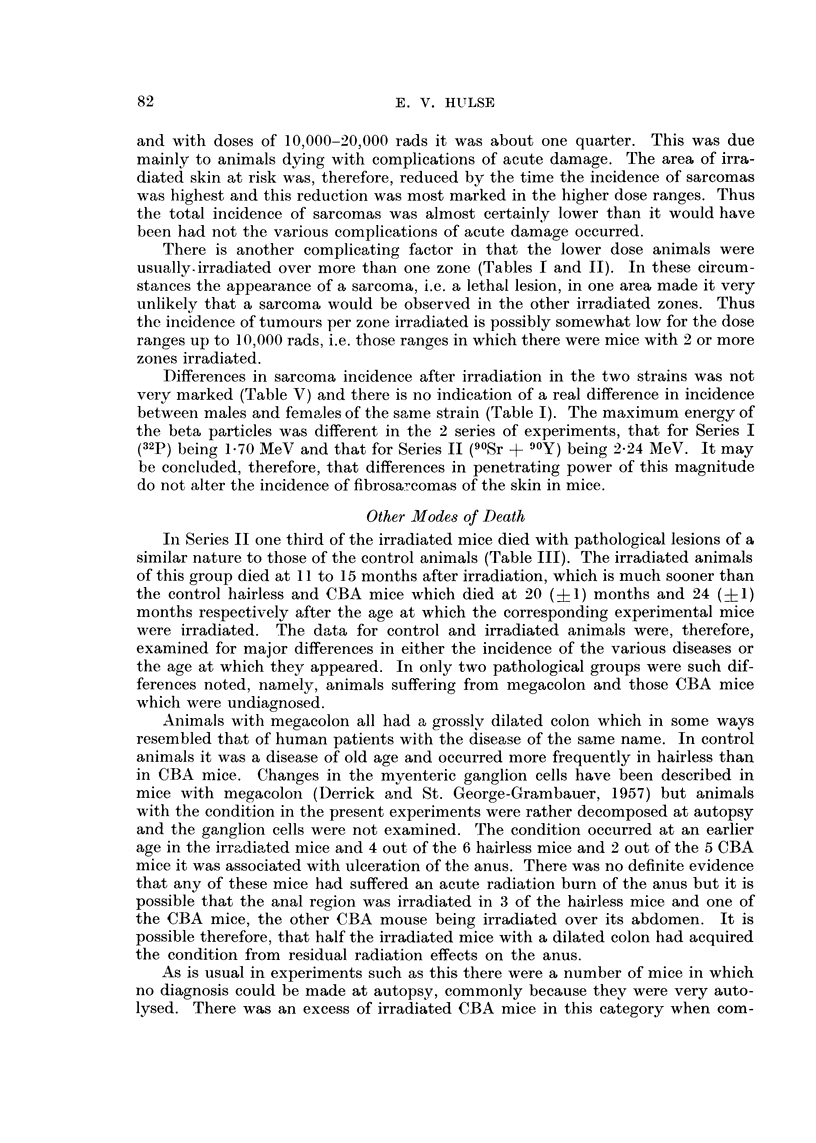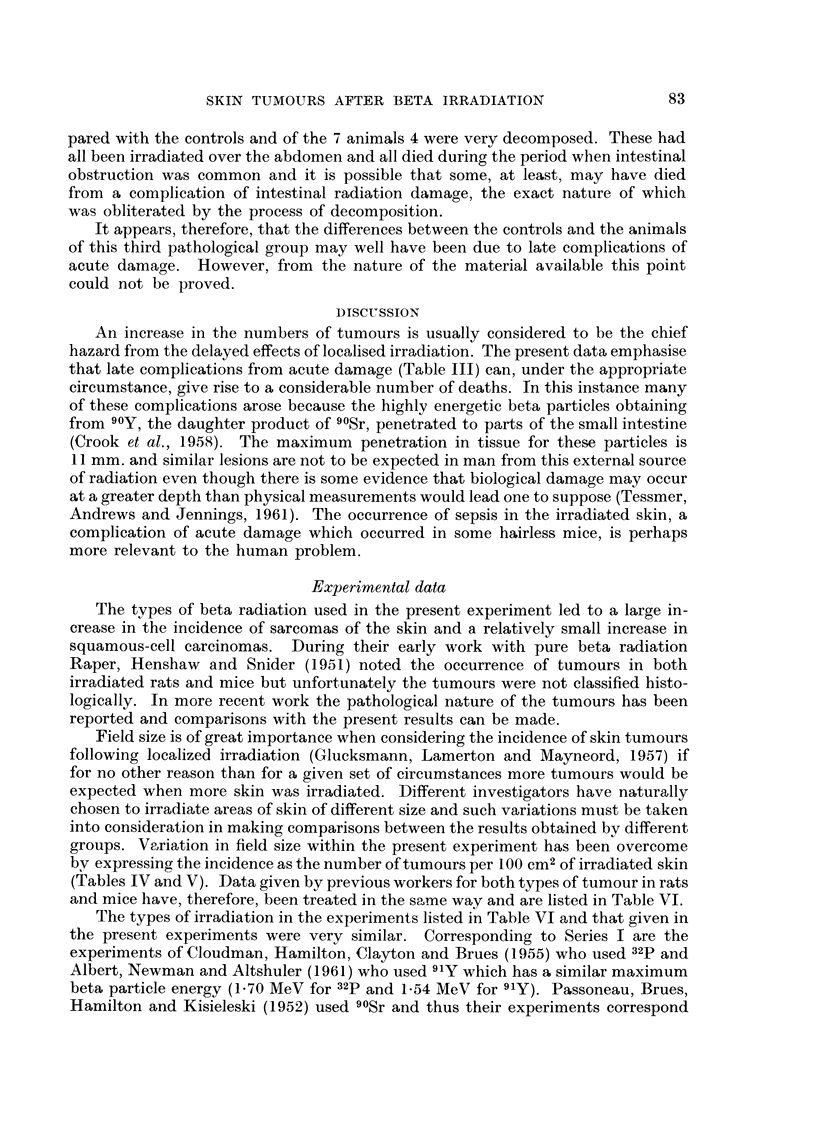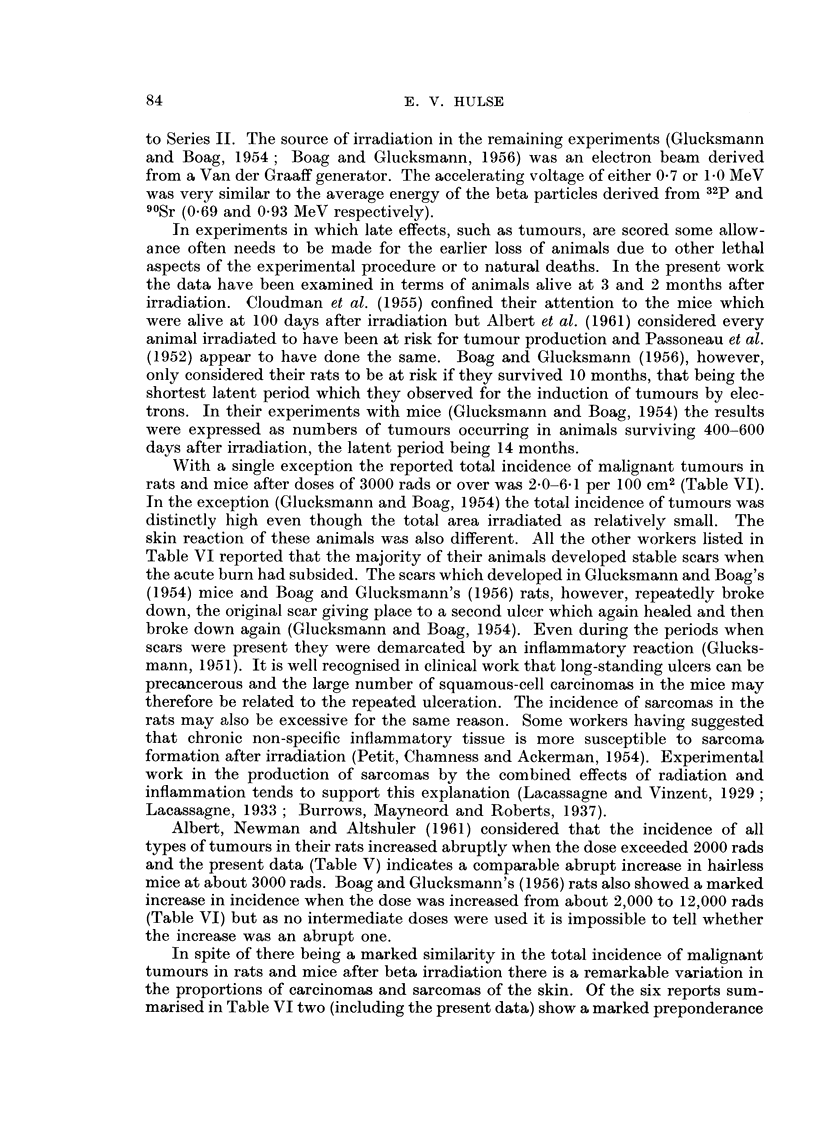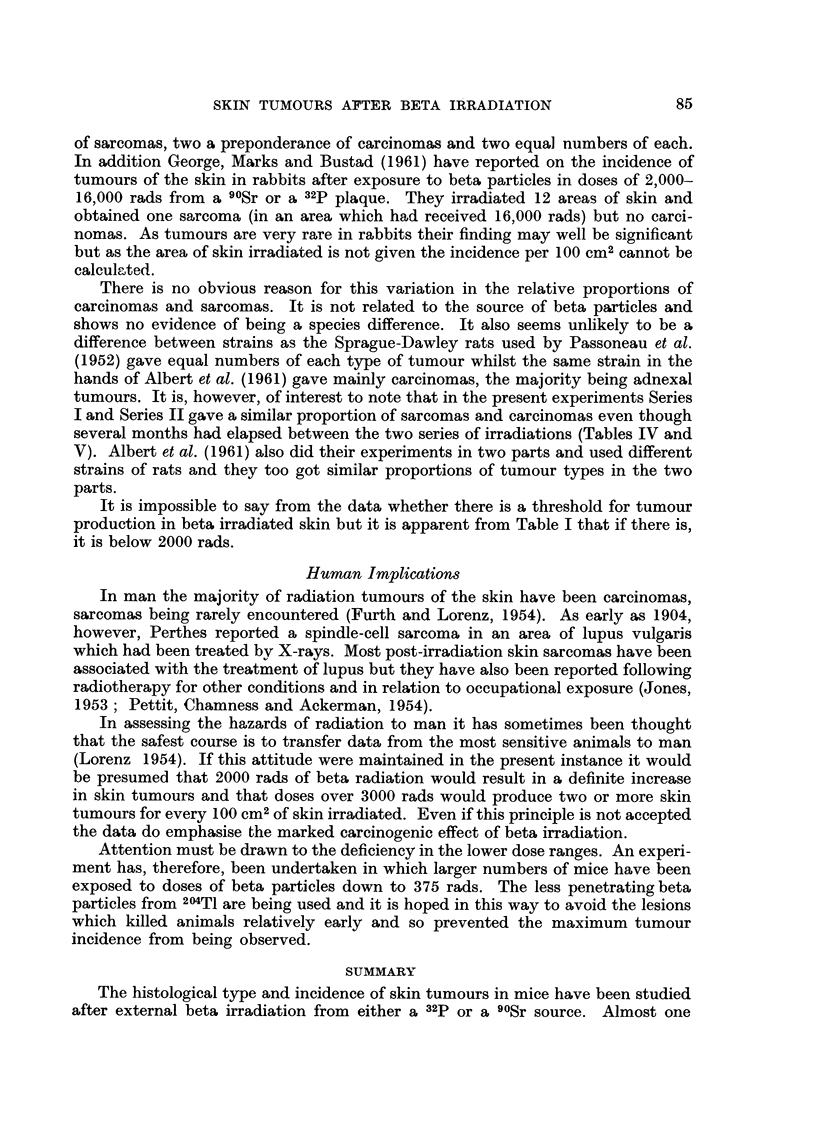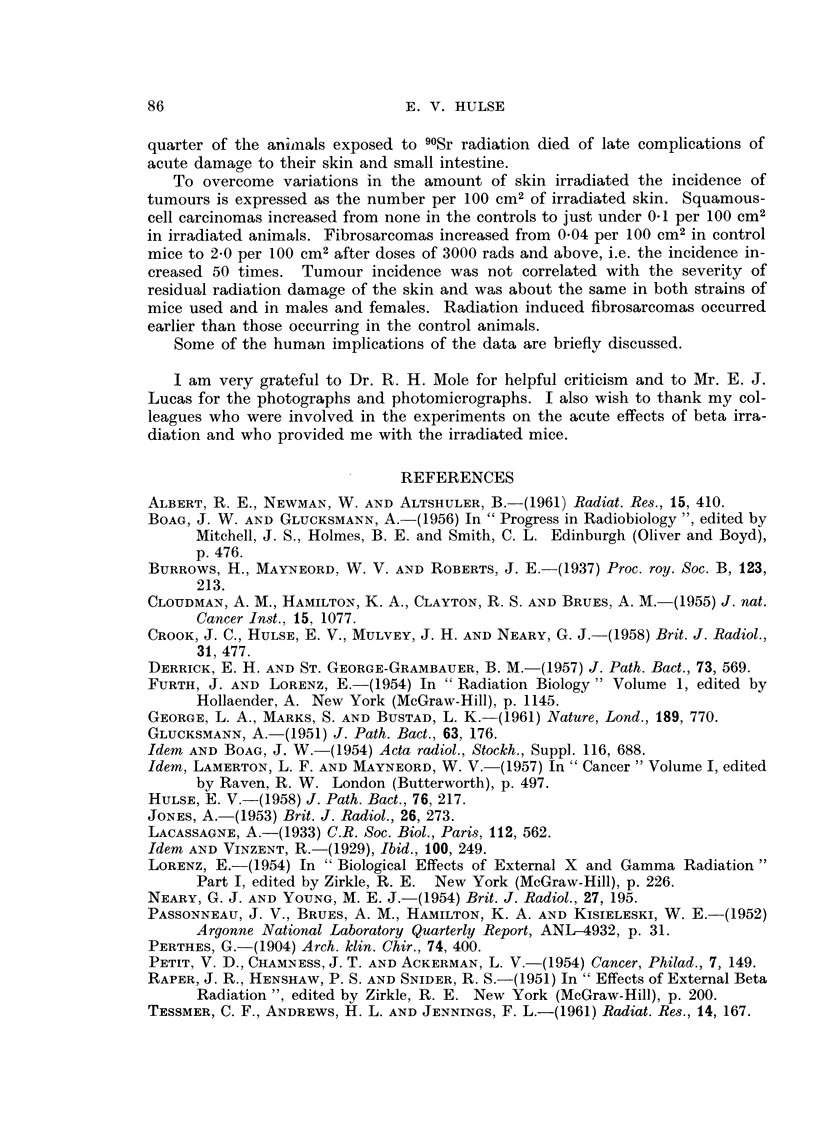# Tumours of the Skin and other Delayed Effects of External Beta Irradiation of Mice Using ^90^Sr and ^32^P

**DOI:** 10.1038/bjc.1962.7

**Published:** 1962-03

**Authors:** E. V. Hulse

## Abstract

**Images:**


### TUMOURS OF THE SKIN AND OTHER DELAYED EFFECTS OF EXTERNAL BETA IRRADIATION OF MICE USING $^{90}Sr$ AND $^{32}P$

E. V. HULSE

*From the Medical Research Council Radiobiological Research Unit, Harwell, Didcot, Berks.*

Received for publication November 24, 1961

THE late effects of any form of ionizing radiation are of interest and importance in relation to occupational exposure and similar hazards. Irradiation of the skin by beta particles is one of the particular hazards of the handling of radioactive isotopes and the opportunity has, therefore, been taken of studying mice which had survived two experiments on the acute effects of beta irradiation. In a preliminary investigation hairless mice were exposed to $^{32}P$ beta rays (Crosland-Taylor, 1954, personal communication); the animals from this experiment will be referred to as Series I. Later both hairless and normally hairy mice were exposed to beta rays from a $^{90}Sr$ source (Crook, Hulse, Mulvey and Neary, 1958) and these animals will be referred to as Series II.

### METHODS

### *Animals*

The animals of Series I were crossbred albino hairless mice, approximately equal numbers of males and females being used. Their mean age at irradiation was 6·3 months (males: 7·4 months; females: 5·1 months). Their weights ranged from 14 g. to 36 g.

In Series II male mice only were used. The mean age of the crossbred albino hairless mice at the time of irradiation was 3·0 months and that of the inbred CBA/H mice 3·2 months. The overall weight-range for Series II was 23 g. to 30 g. All the mice of Series II were free from ectoparasites and thus scratching from that cause was eliminated.

The day of death was recorded for all the animals. In Series I a full pathological examination was made of all animals bearing skin tumours but autopsies were not made on all the non-tumour bearing mice. In Series II a full pathological study was made on all the animals.

### *Radiation procedure*

The apparatus used for irradiating the mice in Series I has already been described in detail by Neary and Young (1954). It consisted essentially of a box lined with panels of phosphorus-Bakelite, the red phosphorus of which had been activated by exposure of the panels to a thermal neutron flux. The box normally gave irradiation to the whole of the body surface but irradiation of only part of the body surface was achieved by placing lead shields between the mouse and the radiation source. The mice were always confined in celluloid tubes 2·5 cm. in diameter and for this experiment either the whole of their body surface, including the head, or a zone of the trunk was exposed. The trunk zones measured 3·8 cm. or 1·9 cm. in length, the shorter zone corresponding to the second or third quarter of the animal and the longer zone to both the second and third quarters, i.e. to the middle half of the mouse (Fig. 1). Normally a very sharp fall off in dose would be expected at the edge of an area exposed to beta rays in this way but, as the irradiation took an hour or longer during which the mouse could move to and fro a little in the celluloid tube, the length of the irradiated zone was slightly greater than that given above and the fall off in dose would also be less sharp.

FIG. 1 (above).—Zones irradiated in Series I. Animals were irradiated (i) over all 4 zones, (ii) over zone " a " or over zone " b " or (iii) over zones " a " + " b ".

FIG. 2 (below).—Zones irradiated in Series II. Animals were irradiated (i) over zones, A, B and C in succession, (ii) over zones A + B, A + C, or B + C or (iii) over zone A, zone B or zone C.

Animals in Series II were irradiated by the method described by Crook *et al.* (1958). $^{90}Sr$ was used as the source of beta rays, being incorporated in an open-ended cylindrical foil 1·4 cm. in length and 2·5 cm. in diameter, the animal again being held within the cylinder by means of a thin-walled celluloid tube similar to that used for Series I. No irradiations of the whole of the body surface or of the head region were carried out in Series II. The zones of irradiation started at a point about 2·5 cm. from the snout and each zone was 1·4 cm. long. The 3 zones which were irradiated are illustrated in Fig. 2. The surface area of each zone was considered to be approximately one-fifth of the total body surface. The mice were exposed to beta radiation (i) over all three zones in succession or (ii) over two zones in succession, either adjacent or separate, or (iii) over one zone only. The positioning of the animals could never be absolutely exact and this, combined with the slight to and fro movement, meant that when adjacent zones were irradiated small areas at the junction of the zones may well have received doses somewhat greater than the dose it was planned to give. As in Series I the radiation dose at the junctions of irradiated and unirradiated skin would decrease somewhat more gradually than if the animal had kept perfectly still.

*Dose of radiation*

The dose of radiation was measured at the inner surface of the celluloid tube, i.e. at the surface in contact with the animal. In Series I the doses ranged from 1,900 rads to 13,000 rads and in Series II from 3,300 rads to 24,000 rads. The actual doses used, the numbers of animals and the number of zones per animal are given in Tables I and II. The dose groups varied a great deal in size partly

TABLE I.—*Number of Animals Exposed to Each Dose of Beta Radiation in Series I and the Numbers of Sarcomas Resulting*

| | | Number of sarcomas/Number of animals | |
|---|---|---|---|
| Number of zones | Dose in rads | Males | Females |
| 4 | 1,900 | 0/6* | 1/5 |
| | 2,300 | 2/6 | 1/4* |
| | 2,800 | 0/1 | 0/1 |
| 2 | 3,700 | 0/1 | 1/3 |
| 1 | 7,450 | 2/6 | 2/7 |
| | 9,300 | 5/9 | 3/8 |
| | 11,200 | 3/3 | 0/3 |
| | 13,000 | 0/4 | 0/2 |
| All zones | — | 12/36 | 8/33 |
| Controls | — | 0/22 | 0/27 |

* One squamous-cell carcinoma occurred in each of these groups, both animals dying at 18 months after irradiation.

because the animals were survivors of acute irradiation experiments. Deaths due to beta-ray burns were found to be increasingly numerous as the area irradiated was increased (Crook *et al.*, 1958), consequently the late effects of the higher doses could be studied only in animals which had been irradiated over one zone. As only small numbers of animals were available at some dose levels subsequent tables (Tables III–V) give dose ranges rather than individual doses. The observations in Series I were commenced at 3 months and in Series II at 2 months after irradiation.

### RESULTS

The acute lesions of the skin have been described previously (Crook *et al.*, 1958). In spite of the high dose of radiation which the skin received the majority of animals developed firm healthy scars. Some, however, retained a few small scabs and some developed a little scaling in the irradiated areas. In a few instances there was gross crusting and indolent ulceration. These late skin changes were much more severe in hairless mice than in CBA mice, a finding which is in keeping with the acute skin changes being more severe in hairless mice (Crook *et al.*, 1958).

TABLE II.—*Number of Animals Exposed to Each Dose of Beta Radiation in Series II and the Numbers of Sarcomas Resulting*

| | hrhr mice | | CBA mice | |
|---|---|---|---|---|
| Number of zones | Dose in rads | Number of sarcomas /Number of animals | Dose in rads | Number of sarcomas /Number of animals |
| 3 | 3,300 | 3/4 | 4,800 | 3/5 |
| | 4,200 | 1/1 | 5,600 | 13/14 |
| | 5,000 | 0/1 | 6,450 | 3/4 |
| | 5,800 | 2/5 | 7,250 | 2/2 |
| | | | 8,000 | 0/1 |
| 2 | 5,000 | 8/12* | 7,200 | 6/10* |
| | 6,250 | 0/7 | 8,450 | 0/12 |
| | 7,500 | 2/4 | 9,650 | 0/1 |
| | 8,750 | 0/1 | | |
| | 10,000 | 1/2 | | |
| 1 | 10,000 | 3/6 | 7,200 | 3/9 |
| | 12,500 | 2/9 | 9,600 | 1/9 |
| | 15,000 | 1/7 | 12,000 | 1/2 |
| | 17,500 | 0/4 | 17,000 | 1/8 |
| | 20,000 | 0/4 | 19,000 | 2/3 |
| | | | 22,000 | 1/1 |
| | | | 24,000 | 1/2 |
| All zones | — | 23/67 | — | 37/83 |
| Controls | — | 2/123 | — | 1/87 |

* One squamous-cell carcinoma occurred in each of these groups, the hrhr mouse dying at 16 months and the CBA mouse at 23 months after irradiation.

None of the animals developed the recurrent ulceration which Glucksmann and Boag (1954) found to be a prominent late effect following exposure of the skin to an electron beam.

The irradiated mice may be divided into 3 groups: (i) animals which developed tumours of the skin, (ii) animals which developed various complications arising from the acute damage produced by the beta irradiation and (iii) animals in which the pathological findings were no different from those of the controls. Of the three groups the mice which developed complications from the acute damage died first and will therefore be considered first.

### *Late Complications of Acute Damage*

Observations on this group are confined to Series II animals because of the incomplete pathological study of Series I. The number of mice affected at different dose ranges and their mean times of death after irradiation are given in Table III.

### *Types of lesion*

High energy beta particles are able to traverse the mouse's abdominal wall and cause acute radiation lesions of the small intestine (Crook *et al.*, 1958). Adhesions between affected segments of the intestine and other viscera or between them and the abdominal wall were fairly frequent as also were localised areas of inflammation in relation to the original intestinal lesions. Some animals died from intestinal obstruction due to these adhesions and others from intraperitoneal abscesses not

TABLE III.—*Number of Animals in the Three Main Pathological Groups and the Time Interval Between Irradiation and Death. Standard Errors Omitted When Number of Animals Was Under Four.*

| Dose in rads | Series | Strain | Total number of mice | Complications of acute damage: Number | Complications of acute damage: Time of death in months (mean ± S.E.) | Tumours of the skin: Number | Tumours of the skin: Time of death in months (mean ± S.E.) | Other diseases: Number | Other diseases: Time of death in months (mean ± S.E.) |
|---|---|---|---|---|---|---|---|---|---|
| Under 3,000 | I | hrhr | 21 | No information | | 6* | 17 ± 1 | 15‡ | 14 ± 1 |
| 3,000–9,990 | I | hrhr | 19 | No information | | 5 | 14 ± 1 | 14‡ | 11 ± 1 |
| | II | hrhr | 35 | 6 | 7 ± 1 | 17† | 15 ± 3 | 12 | 12 ± 1 |
| | II | CBA | 67 | 10 | 8 ± 2 | 32† | 14 ± 1 | 25 | 14 ± 1 |
| 10,000–20,000 | I | hrhr | 29 | No information | | 11 | 14 ± 1 | 18‡ | 11 ± 1 |
| | II | hrhr | 32 | 13 | 6 ± 2 | 7 | 14 ± 1 | 12 | 12 ± 1 |
| | II | CBA | 13 | 5 | 9 ± 2 | 4 | 10 ± 2 | 4 | 15 ± 4 |
| Over 20,000 | II | CBA | 3 | 1 | 7 | 2 | 10 | — | — |

* Includes 2 animals with squamous-cell carcinoma.

† Includes one animal with squamous-cell carcinoma.

‡ Includes animals suffering from complications of acute damage.

only during the first two months after irradiation (Crook *et al.*, 1958; Hulse, 1958) but also subsequently. Over half of the deaths from complications of acute damage were due to this type of lesion.

Irradiation of the perineum, due to the mouse moving in the irradiation tube, led to 3 examples of intestinal obstruction from damage to the anus and one example of urinary obstruction due to damage to the penis. Similar lesions were also seen during the acute stages (Crook *et al.*, 1958).

Severe initial damage to the skin and underlying tissues following irradiation of the thoracic region resulted in occasional examples of (i) constricting scars encircling the chest, (ii) fibrosis of the ventral surface of the heart and pericardium and (iii) a gross deformity of a segment of the sternum giving rise to a spur, the point of which impinged upon the heart. A small number of hairless mice died of abscesses and pyaemia originating from areas of indolent ulceration.

*Incidence and time of death (Table III)*

For doses in the range 3000–9990 rads the incidence of these complications was 15–17 per cent but with doses of 10,000–20,000 rads it was about 40 per cent. The mean time of death did not differ from one dose range to another or from one strain to another and was much less than the mean interval between irradiation and death for animals with skin tumours (Table III). These delayed complications of acute damage, therefore, reduced the numbers of animals at risk for tumour production.

### *Skin Tumours*

In both CBA and hairless mice two kinds of malignant tumours of the skin occurred, namely, squamous-cell carcinoma of the epidermis and fibrosarcoma of the dermis. In neither series did any animal have more than one skin tumour.

It was always possible to decide whether a tumour had occurred in irradiated skin or not because in those animals which had not been irradiated over the whole of their body surface the irradiated area could be identified by the residual scarring in the hairless mice and by the epilation, loss of hair pigment and scarring in the CBA mice. The residual skin changes were no more severe in animals which developed tumours than in animals which did not and no skin tumours occurred amongst the few which developed gross crusting or ulceration.

In both series of experiments the animals which developed tumours were left to die naturally unless the size of the tumour or its degree of ulceration made it necessary to kill the animal. The time interval between irradiation and death was measured to the nearest month and as the tumours in the animals which were killed were very advanced there is little error in using the date of killing as if it were the date of death. All the tumours grew rapidly and most of the animals which developed tumours were dead between one and two months after the tumours were first noticed.

The simple way of expressing tumour incidence as the ratio of the number of animals with a tumour to the total number of animals exposed to a particular dose (Tables I and II) does not allow comparison between groups because of the great variation in the proportion of body surface irradiated. It is more satisfactory to relate the number of tumours to the total area of skin which was irradiated, i.e. " number of tumours per 100 $cm^2$ " of irradiated skin (Tables IV and V). It was presumed that the area of skin irradiated was equal to the surface area of the open-ended cylinder which made the zone i.e. 15 $cm^2$ for Series I and 11 $cm^2$ for Series II. The total area irradiated at any given dose range could then be calculated from the number of mice and the number of zones per mouse irradiated. Contractions of the skin which sometimes occurred in relation to a radiation scar were disregarded in the calculations and the area of skin " at risk " was always presumed to be equal to the area of skin which was irradiated. When calculating the area of skin at risk in the control mice it was presumed that the available surface area of each mouse was equal to that of the maximum area irradiated in any one animal of the series, e.g. 4 zones in Series I and 3 zones in Series II. Similarly control information is available from the unirradiated zones of irradiated mice (Table V).

### *Squamous-cell Carcinomas*

Four squamous-cell carcinomas of the skin appeared amongst 219 irradiated mice but none occurred amongst 259 control animals. All the tumours occurred in irradiated skin. Details of the doses of radiation at which they occurred are given in Tables I and II and the incidence per 100 $cm^2$ of irradiated skin is given in Table IV. When the overall incidences in irradiated and control mice are compared by Fisher and Yates' exact test there is a statistically significant increase in the irradiated animals ($P = 0{\cdot}04$ using the number of tumours per mouse and $P = 0{\cdot}0003$ using the number of tumours per unit area of skin at risk).

The squamous-cell carcinomas appeared late in life, after the majority of the mice were dead (cf. time of death of mice with carcinomas given in Tables I and II with the mean time of death from other causes given in Table III). Thus the observed incidence may be an underestimate. The earliest time at which an animal died with a squamous-cell carcinoma was 16 months after irradiation and

TABLE IV.—*Number of Squamous-cell Carcinomas of the Skin, Area of Skin at Risk and Incidence of Squamous-cell Carcinomas Expressed as Number of Tumours per* 100 $cm^2$ *of Skin. No Tumours of this Type Appeared in Unirradiated Skin.*

| | | | | Skin at risk during whole of experiment | | Skin at risk at 16 months after irradiation* | |
|---|---|---|---|---|---|---|---|
| Dose in rads | Series | Strain | Number of carcinomas | Area in $cm^2$ | Carcinomas per 100 $cm^2$ | Area in $cm^2$ | Carcinomas per 100 $cm^2$ |
| 1,900–13,000 | I | hrhr | 2 | 2,130 | 0·09 | 735 | 0·3 |
| 3,300–20,000 | II | hrhr | 1 | 1,265 | 0·08 | 352 | 0·3 |
| 4,800–24,000 | II | CBA | 1 | 1,738 | 0·06 | 484 | 0·2 |

* The earliest squamous-cell carcinoma death occurred at 16 months after irradiation.

if the calculations are made using the area at risk at that time the incidence is about four times greater (Table IV).

### *Fibrosarcomas*

There was a marked increase in the incidence of fibrosarcomas of the skin in irradiated mice (Tables I and II).

*Gross appearances*

The tumours started as small masses firmly attached to the overlying epidermis. As they increased in size most of them became ulcerated. They all occurred in irradiated areas and none was found at the junction of irradiated and non-irradiated tissue. Typical appearances are shown in Fig. 3 and 4. The animal in Fig. 3 was irradiated over 3 zones and developed a tumour in an area which was completely epilated. The early tumour illustrated in Fig. 4 occurred in an animal irradiated over one zone and appears to have arisen in the centre of the linear scar which developed after the initial radiation effects had subsided. The tumours frequently spread locally by direct extension but distant metastases were seen in only one animal.

One of the 67 irradiated hairless mice of Series II and one of the 83 irradiated CBA mice developed a fibrosarcoma of the orbit, i.e. well outside the irradiated zone and these tumours are not included in the analysis of the 60 other sarcomas in the irradiated animals of Series II.

---

EXPLANATION OF PLATE

FIG. 3. —CBA mouse showing fibrosarcoma of skin, with early ulceration, 18 months after receiving 4800 rads of beta irradiation over zones A, B and C (Fig. 2).

FIG. 4.—Hairless mouse showing an early fibrosarcoma in the linear scar in zone C. Photographed 14 months after 7500 rads of beta irradiation to zones A and C.

FIG. 5.—Well differentiated fibrosarcoma of skin from a hairless mouse of Series II which died 10 months after a dose of 5000 rads to 2 zones. Haematoxylin and Eosin. ×120.

FIG. 6.—Area of an otherwise well differentiated fibrosarcoma showing cells with large bizarre shaped nuclei. From a CBA mouse which died 11 months after 7200 rads over 3 zones. Haematoxylin and Eosin. ×340.

FIG. 7.—Anaplastic fibrosarcoma from a CBA mouse which died 7 months after 12,000 rads to one zone. Haematoxylin and Eosin. × 120.

FIG. 8.—Fibrosarcoma with areas of giant cells. CBA mouse which died 7 months after 5600 rads to 3 zones. Haematoxylin and Eosin. ×120.

3

5

4

6

7

8

*Histology*

All the tumours were very cellular and histologically were shown to be fibrosarcomas. Many were well differentiated (Fig. 5) but it was not unusual to find scattered cells with bizarre nuclei even in well differentiated tumours (Fig. 6). Some tumours were more anaplastic (Fig. 7) and a small proportion showed large numbers of cells with giant nuclei (Fig. 8). However anaplastic a tumour appeared, it was always possible to find some areas which indicated its fibrous nature. Mild inflammatory changes were present in those tumours which were ulcerated. Each tumour was situated between the epidermis and the panniculus carnosus and none of the tumours were encapsulated. They all showed evidence of infiltration into surrounding connective tissue and the majority had also infiltrated into or right through the panniculus.

*Mean time of death*

The mean time intervals between irradiation and death for animals with skin tumours are given in Table III. So few of these tumours were squamous-cell carcinomas that the times given in Table III are identical to the mean times between irradiation and death for animals with fibrosarcomas of the skin. The 2 control hairless mice which developed fibrosarcomas of the skin died at 15 and 18 months and the control CBA mouse at 24 months after the age at which they would have been irradiated. When these times are compared with those in Table III it is apparent that fibrosarcomas occurring in mice given 3000 rads or over resulted in the animals dying sooner than the controls and there is also evidence that the higher the dose of radiation the sooner death from fibrosarcoma of the skin occurred.

The minimum time from irradiation to death due to fibrosarcoma of the skin was 5 months. It is in keeping with the trends noted above that this CBA mouse, which was allowed to die naturally, received a dose of 24,000 rads, i.e. the highest dose used.

*Incidence of fibrosarcomas of the skin*

In the hairless mice there was a very definite increase in incidence with doses of under 3000 rads (Tables I and V). With doses of 3000–9990 rads there was a further marked increase in incidence but with doses of 10,000–20,000 rads there was no convincing evidence of a further change in incidence (Table V). The CBA mice, however, showed a progressive increase in incidence over the 3 dose ranges listed in Table V (about 80 times the control after 10,000–20,000 rads and about 170 times the control when the dose was over 20,000 rads).

The mean time from irradiation to death from causes other than skin tumour was almost always less than the mean time to death of animals with skin tumours (Table III). In order to make some allowance for this the tumour incidence is shown separately for mice dying before 14 months and after 13 months (Table V). This particular time was chosen because it was the mean time of death of all tumour bearing animals. In nearly every case the incidence was highest in the older animals and in one instance (Series I ; 10,000–20,000 rads) the incidence amongst the older animals was nearly three times that derived from considering all the animals at risk in the group.

Table V also indicates the marked reduction in the area of skin at risk at 14 months after irradiation : with doses of 3000–9990 rads it was down to a half

TABLE V.—*Number of Sarcomas of Skin, Area of Irradiated and Unirradiated Skin at Risk and the Incidence of Sarcomas Expressed as Number of Tumours per 100 cm² of Skin.*

| Dose in rads | Series | Strain | All animals: Number of sarcomas | All animals: Area at risk (in cm²) | All animals: Sarcomas per 100 cm² | Animals dying between 5 and 13 months after irradiation*: Number of sarcomas | Area at risk (in cm²) | Sarcomas per 100 cm² | Animals dying at 14 months after irradiation or later*: Number of sarcomas | Area at risk (in cm²) | Sarcomas per 100 cm² |
|---|---|---|---|---|---|---|---|---|---|---|---|
| Under 3,000 | I | hrhr | 4 | 1,380 | 0·3 | 1 | 1,380 | 0·07 | 3 | 780 | 0·4 |
| 3,000–9,990 | I | hrhr | 13 | 570 | 2·3 | 5 | 555 | 0·9 | 8 | 225 | 3·6 |
| | II | hrhr | 16 | 891 | 1·8 | 4 | 869 | 0·5 | 12 | 473 | 2·5 |
| | II | CBA | 31 | 1,562 | 2·0 | 15 | 1,463 | 1·0 | 16 | 660 | 2·4 |
| 10,000–20,000 | I | hrhr | 3 | 180 | 1·7 | 1 | 180 | 0·6 | 2 | 45 | 4·5 |
| | II | hrhr | 7 | 374 | 1·9 | 3 | 341 | 0·9 | 4 | 110 | 3·7 |
| | II | CBA | 4 | 143 | 2·8 | 4 | 132 | 3·0 | 0 | 33 | 0 |
| Over 20,000 | II | CBA | 2 | 33 | 6·1 | 1 | 33 | 3·0 | 1 | 11 | 9·1 |
| Unirradiated mice | I | hrhr | 0 | 2,940 | 0 | 0 | 2,940 | 0 | 0 | 1,980 | 0 |
| | II | hrhr | 2 | 4,059 | 0·05 | 0 | 4,059 | 0 | 2 | 3,696 | 0·05 |
| | II | CBA | 1 | 2,871 | 0·04 | 0 | 2,871 | 0 | 1 | 2,739 | 0·04 |
| Unirradiated zones in irradiated mice | I | hrhr | 0 | 2,010 | 0 | 0 | 2,010 | 0 | 0 | 750 | 0 |
| | II | hrhr | 0 | 946 | 0 | 0 | 869 | 0 | 0 | 308 | 0 |
| | II | CBA | 0 | 1,001 | 0 | 0 | 913 | 0 | 0 | 484 | 0 |

* The earliest sarcoma death occurred at 5 months and the mean time to death of sarcoma bearing animals was 14 months after irradiation

TABLE VI.—*Incidence of Malignant Tumours of the Skin in the Present Experiments Compared with the Incidences Obtained by Other Workers Experimenting with Beta Particles or Electrons.*

| | Present data | | Glucksmann & Boag (1954) | Cloudman *et al.* (1955) | Passoneau *et al.* (1952) | Boag & Glucksmann (1956) | | Albert *et al.* (1961)† | |
|---|---|---|---|---|---|---|---|---|---|
| Species | mice | mice | mice | mice | rats | rats | rats | rats | rats |
| Strain | hrhr | hrhr & CBA | C57 | CF-1 | Sprague-Dawley | Black-hooded | Black-hooded | Sprague-Dawley & Holtzmann | Sprague-Dawley & Holtzmann |
| Dose range (in rads) | 1,900–2,800 | 3,300–24,000 | 8,000 | 4,600–9,300 | 5,500–8,200 | 2,000 or 2,300 | 12,000 | 230–2,500 | 3,750–10,000 |
| Individual area irradiated (cm$^2$) | 11 or 15 | 11 or 15 | 0·8 | 4 | 35 | 4·9 or 10 | 4·9 | 32 or 35 | 32 or 35 |
| Total area irradiated in all animals (cm$^2$) | 1,380 | 3,753 | 21 | 420 | 5,565 | 338* | 245* | 6,340–6,930 | 2,850–3,115 |
| Number of carcinomas | 2 | 2 | 4 | 4 | 71 | 1 | 1 | 43 | 168 |
| Number of sarcomas | 4 | 76 | 0 | 4 | 73 | 5 | 14 | 5 | 4 |
| Malignant tumours per 100 cm$^2$ of irradiated skin | 0·4 | 2·1 | 19 | 2·0 | 2·6 | 1·8 | 6·1 | 0·8 | 5·8 |
| Carcinomas per 100 cm$^2$ of irradiated skin | 0·1 | 0·05 | 19 | 1·0 | 1·3 | 0·3 | 0·4 | 0·7 | 5·7 |
| Sarcomas per 100 cm$^2$ of irradiated skin | 0·3 | 2·0 | 0 | 1·0 | 1·3 | 1·5 | 5·7 | 0·08 | 0·1 |

* Including 24 animals irradiated over 2 zones, one zone receiving 2,000 rads and the other 12,000 rads.

† In extracting the data from Albert *et al.* (1961) tumours listed as " adnexal tumours " have been presumed to be carcinomas and those listed as " connective tissue tumours " as sarcomas. Tumours in which there was no pathological examination are not included.

and with doses of 10,000–20,000 rads it was about one quarter. This was due mainly to animals dying with complications of acute damage. The area of irradiated skin at risk was, therefore, reduced by the time the incidence of sarcomas was highest and this reduction was most marked in the higher dose ranges. Thus the total incidence of sarcomas was almost certainly lower than it would have been had not the various complications of acute damage occurred.

There is another complicating factor in that the lower dose animals were usually irradiated over more than one zone (Tables I and II). In these circumstances the appearance of a sarcoma, i.e. a lethal lesion, in one area made it very unlikely that a sarcoma would be observed in the other irradiated zones. Thus the incidence of tumours per zone irradiated is possibly somewhat low for the dose ranges up to 10,000 rads, i.e. those ranges in which there were mice with 2 or more zones irradiated.

Differences in sarcoma incidence after irradiation in the two strains was not very marked (Table V) and there is no indication of a real difference in incidence between males and females of the same strain (Table I). The maximum energy of the beta particles was different in the 2 series of experiments, that for Series I ($^{32}P$) being 1·70 MeV and that for Series II ($^{90}Sr + ^{90}Y$) being 2·24 MeV. It may be concluded, therefore, that differences in penetrating power of this magnitude do not alter the incidence of fibrosarcomas of the skin in mice.

### *Other Modes of Death*

In Series II one third of the irradiated mice died with pathological lesions of a similar nature to those of the control animals (Table III). The irradiated animals of this group died at 11 to 15 months after irradiation, which is much sooner than the control hairless and CBA mice which died at 20 ($\pm 1$) months and 24 ($\pm 1$) months respectively after the age at which the corresponding experimental mice were irradiated. The data for control and irradiated animals were, therefore, examined for major differences in either the incidence of the various diseases or the age at which they appeared. In only two pathological groups were such differences noted, namely, animals suffering from megacolon and those CBA mice which were undiagnosed.

Animals with megacolon all had a grossly dilated colon which in some ways resembled that of human patients with the disease of the same name. In control animals it was a disease of old age and occurred more frequently in hairless than in CBA mice. Changes in the myenteric ganglion cells have been described in mice with megacolon (Derrick and St. George-Grambauer, 1957) but animals with the condition in the present experiments were rather decomposed at autopsy and the ganglion cells were not examined. The condition occurred at an earlier age in the irradiated mice and 4 out of the 6 hairless mice and 2 out of the 5 CBA mice it was associated with ulceration of the anus. There was no definite evidence that any of these mice had suffered an acute radiation burn of the anus but it is possible that the anal region was irradiated in 3 of the hairless mice and one of the CBA mice, the other CBA mouse being irradiated over its abdomen. It is possible therefore, that half the irradiated mice with a dilated colon had acquired the condition from residual radiation effects on the anus.

As is usual in experiments such as this there were a number of mice in which no diagnosis could be made at autopsy, commonly because they were very autolysed. There was an excess of irradiated CBA mice in this category when compared with the controls and of the 7 animals 4 were very decomposed. These had all been irradiated over the abdomen and all died during the period when intestinal obstruction was common and it is possible that some, at least, may have died from a complication of intestinal radiation damage, the exact nature of which was obliterated by the process of decomposition.

It appears, therefore, that the differences between the controls and the animals of this third pathological group may well have been due to late complications of acute damage. However, from the nature of the material available this point could not be proved.

### DISCUSSION

An increase in the numbers of tumours is usually considered to be the chief hazard from the delayed effects of localised irradiation. The present data emphasise that late complications from acute damage (Table III) can, under the appropriate circumstance, give rise to a considerable number of deaths. In this instance many of these complications arose because the highly energetic beta particles obtaining from $^{90}$Y, the daughter product of $^{90}$Sr, penetrated to parts of the small intestine (Crook *et al.*, 1958). The maximum penetration in tissue for these particles is 11 mm. and similar lesions are not to be expected in man from this external source of radiation even though there is some evidence that biological damage may occur at a greater depth than physical measurements would lead one to suppose (Tessmer, Andrews and Jennings, 1961). The occurrence of sepsis in the irradiated skin, a complication of acute damage which occurred in some hairless mice, is perhaps more relevant to the human problem.

### *Experimental data*

The types of beta radiation used in the present experiment led to a large increase in the incidence of sarcomas of the skin and a relatively small increase in squamous-cell carcinomas. During their early work with pure beta radiation Raper, Henshaw and Snider (1951) noted the occurrence of tumours in both irradiated rats and mice but unfortunately the tumours were not classified histologically. In more recent work the pathological nature of the tumours has been reported and comparisons with the present results can be made.

Field size is of great importance when considering the incidence of skin tumours following localized irradiation (Glucksmann, Lamerton and Mayneord, 1957) if for no other reason than for a given set of circumstances more tumours would be expected when more skin was irradiated. Different investigators have naturally chosen to irradiate areas of skin of different size and such variations must be taken into consideration in making comparisons between the results obtained by different groups. Variation in field size within the present experiment has been overcome by expressing the incidence as the number of tumours per 100 cm$^2$ of irradiated skin (Tables IV and V). Data given by previous workers for both types of tumour in rats and mice have, therefore, been treated in the same way and are listed in Table VI.

The types of irradiation in the experiments listed in Table VI and that given in the present experiments were very similar. Corresponding to Series I are the experiments of Cloudman, Hamilton, Clayton and Brues (1955) who used $^{32}$P and Albert, Newman and Altshuler (1961) who used $^{91}$Y which has a similar maximum beta particle energy (1·70 MeV for $^{32}$P and 1·54 MeV for $^{91}$Y). Passoneau, Brues, Hamilton and Kisieleski (1952) used $^{90}$Sr and thus their experiments correspond to Series II. The source of irradiation in the remaining experiments (Glucksmann and Boag, 1954 ; Boag and Glucksmann, 1956) was an electron beam derived from a Van der Graaff generator. The accelerating voltage of either 0·7 or 1·0 MeV was very similar to the average energy of the beta particles derived from $^{32}$P and $^{90}$Sr (0·69 and 0·93 MeV respectively).

In experiments in which late effects, such as tumours, are scored some allowance often needs to be made for the earlier loss of animals due to other lethal aspects of the experimental procedure or to natural deaths. In the present work the data have been examined in terms of animals alive at 3 and 2 months after irradiation. Cloudman *et al.* (1955) confined their attention to the mice which were alive at 100 days after irradiation but Albert *et al.* (1961) considered every animal irradiated to have been at risk for tumour production and Passoneau *et al.* (1952) appear to have done the same. Boag and Glucksmann (1956), however, only considered their rats to be at risk if they survived 10 months, that being the shortest latent period which they observed for the induction of tumours by electrons. In their experiments with mice (Glucksmann and Boag, 1954) the results were expressed as numbers of tumours occurring in animals surviving 400–600 days after irradiation, the latent period being 14 months.

With a single exception the reported total incidence of malignant tumours in rats and mice after doses of 3000 rads or over was 2·0–6·1 per 100 cm$^2$ (Table VI). In the exception (Glucksmann and Boag, 1954) the total incidence of tumours was distinctly high even though the total area irradiated as relatively small. The skin reaction of these animals was also different. All the other workers listed in Table VI reported that the majority of their animals developed stable scars when the acute burn had subsided. The scars which developed in Glucksmann and Boag's (1954) mice and Boag and Glucksmann's (1956) rats, however, repeatedly broke down, the original scar giving place to a second ulcer which again healed and then broke down again (Glucksmann and Boag, 1954). Even during the periods when scars were present they were demarcated by an inflammatory reaction (Glucksmann, 1951). It is well recognised in clinical work that long-standing ulcers can be precancerous and the large number of squamous-cell carcinomas in the mice may therefore be related to the repeated ulceration. The incidence of sarcomas in the rats may also be excessive for the same reason. Some workers having suggested that chronic non-specific inflammatory tissue is more susceptible to sarcoma formation after irradiation (Petit, Chamness and Ackerman, 1954). Experimental work in the production of sarcomas by the combined effects of radiation and inflammation tends to support this explanation (Lacassagne and Vinzent, 1929 ; Lacassagne, 1933 ; Burrows, Mayneord and Roberts, 1937).

Albert, Newman and Altshuler (1961) considered that the incidence of all types of tumours in their rats increased abruptly when the dose exceeded 2000 rads and the present data (Table V) indicates a comparable abrupt increase in hairless mice at about 3000 rads. Boag and Glucksmann's (1956) rats also showed a marked increase in incidence when the dose was increased from about 2,000 to 12,000 rads (Table VI) but as no intermediate doses were used it is impossible to tell whether the increase was an abrupt one.

In spite of there being a marked similarity in the total incidence of malignant tumours in rats and mice after beta irradiation there is a remarkable variation in the proportions of carcinomas and sarcomas of the skin. Of the six reports summarised in Table VI two (including the present data) show a marked preponderance of sarcomas, two a preponderance of carcinomas and two equal numbers of each. In addition George, Marks and Bustad (1961) have reported on the incidence of tumours of the skin in rabbits after exposure to beta particles in doses of 2,000–16,000 rads from a $^{90}Sr$ or a $^{32}P$ plaque. They irradiated 12 areas of skin and obtained one sarcoma (in an area which had received 16,000 rads) but no carcinomas. As tumours are very rare in rabbits their finding may well be significant but as the area of skin irradiated is not given the incidence per 100 $cm^2$ cannot be calculated.

There is no obvious reason for this variation in the relative proportions of carcinomas and sarcomas. It is not related to the source of beta particles and shows no evidence of being a species difference. It also seems unlikely to be a difference between strains as the Sprague-Dawley rats used by Passoneau *et al.* (1952) gave equal numbers of each type of tumour whilst the same strain in the hands of Albert *et al.* (1961) gave mainly carcinomas, the majority being adnexal tumours. It is, however, of interest to note that in the present experiments Series I and Series II gave a similar proportion of sarcomas and carcinomas even though several months had elapsed between the two series of irradiations (Tables IV and V). Albert *et al.* (1961) also did their experiments in two parts and used different strains of rats and they too got similar proportions of tumour types in the two parts.

It is impossible to say from the data whether there is a threshold for tumour production in beta irradiated skin but it is apparent from Table I that if there is, it is below 2000 rads.

### *Human Implications*

In man the majority of radiation tumours of the skin have been carcinomas, sarcomas being rarely encountered (Furth and Lorenz, 1954). As early as 1904, however, Perthes reported a spindle-cell sarcoma in an area of lupus vulgaris which had been treated by X-rays. Most post-irradiation skin sarcomas have been associated with the treatment of lupus but they have also been reported following radiotherapy for other conditions and in relation to occupational exposure (Jones, 1953 ; Pettit, Chamness and Ackerman, 1954).

In assessing the hazards of radiation to man it has sometimes been thought that the safest course is to transfer data from the most sensitive animals to man (Lorenz 1954). If this attitude were maintained in the present instance it would be presumed that 2000 rads of beta radiation would result in a definite increase in skin tumours and that doses over 3000 rads would produce two or more skin tumours for every 100 $cm^2$ of skin irradiated. Even if this principle is not accepted the data do emphasise the marked carcinogenic effect of beta irradiation.

Attention must be drawn to the deficiency in the lower dose ranges. An experiment has, therefore, been undertaken in which larger numbers of mice have been exposed to doses of beta particles down to 375 rads. The less penetrating beta particles from $^{204}Tl$ are being used and it is hoped in this way to avoid the lesions which killed animals relatively early and so prevented the maximum tumour incidence from being observed.

### SUMMARY

The histological type and incidence of skin tumours in mice have been studied after external beta irradiation from either a $^{32}P$ or a $^{90}Sr$ source. Almost one quarter of the animals exposed to $^{90}Sr$ radiation died of late complications of acute damage to their skin and small intestine.

To overcome variations in the amount of skin irradiated the incidence of tumours is expressed as the number per 100 cm$^2$ of irradiated skin. Squamous-cell carcinomas increased from none in the controls to just under 0·1 per 100 cm$^2$ in irradiated animals. Fibrosarcomas increased from 0·04 per 100 cm$^2$ in control mice to 2·0 per 100 cm$^2$ after doses of 3000 rads and above, i.e. the incidence increased 50 times. Tumour incidence was not correlated with the severity of residual radiation damage of the skin and was about the same in both strains of mice used and in males and females. Radiation induced fibrosarcomas occurred earlier than those occurring in the control animals.

Some of the human implications of the data are briefly discussed.

I am very grateful to Dr. R. H. Mole for helpful criticism and to Mr. E. J. Lucas for the photographs and photomicrographs. I also wish to thank my colleagues who were involved in the experiments on the acute effects of beta irradiation and who provided me with the irradiated mice.

### REFERENCES

Albert, R. E., Newman, W. and Altshuler, B.—(1961) *Radiat. Res.*, **15**, 410.
Boag, J. W. and Glucksmann, A.—(1956) In " Progress in Radiobiology ", edited by Mitchell, J. S., Holmes, B. E. and Smith, C. L. Edinburgh (Oliver and Boyd), p. 476.
Burrows, H., Mayneord, W. V. and Roberts, J. E.—(1937) *Proc. roy. Soc.* B, **123**, 213.
Cloudman, A. M., Hamilton, K. A., Clayton, R. S. and Brues, A. M.—(1955) *J. nat. Cancer Inst.*, **15**, 1077.
Crook, J. C., Hulse, E. V., Mulvey, J. H. and Neary, G. J.—(1958) *Brit. J. Radiol.*, **31**, 477.
Derrick, E. H. and St. George-Grambauer, B. M.—(1957) *J. Path. Bact.*, **73**, 569.
Furth, J. and Lorenz, E.—(1954) In " Radiation Biology " Volume 1, edited by Hollaender, A. New York (McGraw-Hill), p. 1145.
George, L. A., Marks, S. and Bustad, L. K.—(1961) *Nature, Lond.*, **189**, 770.
Glucksmann, A.—(1951) *J. Path. Bact.*, **63**, 176.
*Idem* and Boag, J. W.—(1954) *Acta radiol., Stockh.*, Suppl. 116, 688.
*Idem*, Lamerton, L. F. and Mayneord, W. V.—(1957) In " Cancer " Volume I, edited by Raven, R. W. London (Butterworth), p. 497.
Hulse, E. V.—(1958) *J. Path. Bact.*, **76**, 217.
Jones, A.—(1953) *Brit. J. Radiol.*, **26**, 273.
Lacassagne, A.—(1933) *C.R. Soc. Biol., Paris*, **112**, 562.
*Idem* and Vinzent, R.—(1929), *Ibid.*, **100**, 249.
Lorenz, E.—(1954) In " Biological Effects of External X and Gamma Radiation " Part I, edited by Zirkle, R. E. New York (McGraw-Hill), p. 226.
Neary, G. J. and Young, M. E. J.—(1954) *Brit. J. Radiol.*, **27**, 195.
Passonneau, J. V., Brues, A. M., Hamilton, K. A. and Kisieleski, W. E.—(1952) *Argonne National Laboratory Quarterly Report*, ANL-4932, p. 31.
Perthes, G.—(1904) *Arch. klin. Chir.*, **74**, 400.
Petit, V. D., Chamness, J. T. and Ackerman, L. V.—(1954) *Cancer, Philad.*, **7**, 149.
Raper, J. R., Henshaw, P. S. and Snider, R. S.—(1951) In " Effects of External Beta Radiation ", edited by Zirkle, R. E. New York (McGraw-Hill), p. 200.
Tessmer, C. F., Andrews, H. L. and Jennings, F. L.—(1961) *Radiat. Res.*, **14**, 167.